# Effects of different blood storage conditions on leukocytes cell population data using the Sysmex XN hematological analyzer

**DOI:** 10.11613/BM.2026.020704

**Published:** 2026-04-15

**Authors:** Vincenzo Roccaforte, Rossella Panella, Massimo Daves, Antonella De Martino, Erika Jani, Giovanni Sabbatini, Pierfrancesco Agostini, Floriana De Filippi, Paolo Formenti, Giuseppe Lippi, Matteo Vidali, Angelo Pezzi, Stefano Pastori

**Affiliations:** 1 S.C. Analisi Chimico Cliniche e Microbiologiche, ASST Nord Milano, Ospedale Bassini, Cinisello Balsamo, Italy; 2 UOSVD Patologia Clinica, ASL Bari, Ospedale San Paolo, Bari, Italy; 3 Laboratory of Clinical Biochemistry (SABES-ASDAA), Hospital of Bolzano, Bolzano, Italy; 4 S.C. Anestesia, Rianimazione e Terapia Intensiva, ASST Nord Milano, Ospedale Bassini, Cinisello Balsamo, Italy; 5 Section of Clinical Biochemistry, University of Verona, Verona, Italy; 6 Clinical Pathology Unit, Fondazione IRCCS Ca’ Granda Ospedale Maggiore Policlinico, Milan, Italy

**Keywords:** cell population data (CPD), Sysmex, sample stability, experimental hematology parameters, pre-analytical variability

## Abstract

**Introduction:**

The aim of this study was to investigate the stability of leukocyte cell population data (CPD) assessed on Sysmex XN-3000 hematological analyzer in samples stored for up to 24 h at different temperatures.

**Materials and methods:**

Whole-blood samples from 25 donors were analyzed for 18 CPD parameters at baseline and after storage at room temperature (RT), 4 °C, or 37 °C for 3, 6, 12, and 24 h. Stability was assessed using three complementary approaches: paired Wilcoxon tests with Bonferroni correction, percent difference between medians > 10%, and *per*-sample percent change > 10% in ≥ 2 samples. In addition, the European Federation of Clinical Chemistry and Laboratory Medicine (EFLM) stability protocol was applied, modelling percent differences over time using linear regression through the origin to estimate the rate of change and the stability limit (SL), defined as the time required to exceed a maximum permissible error of 10%.

**Results:**

According to predefined stability criteria, several CPD parameters exceeded stability limits within 24 h, particularly under storage at 37 °C. Parameters related to neutrophil, lymphocyte and monocyte complexity and size (*e.g.*, neutrophils complexity (NE-SSC), neutrophils size (NE-FSC), monocytes complexity (MO-X), lymphocytes complexity (LY-X); where SSC = side scatter, FSC = forward scatter) showed slow rates of change with estimated stability limits beyond 24 h across storage conditions. In contrast, fluorescence- and dispersion-related parameters (*e.g*., neutrophils fluorescence intensity (NE-SFL), width of dispersion of neutrophils complexity (NE-WX), width of dispersion of lymphocytes fluorescence (LY-WY), width of dispersion of monocytes size (MO-WZ); where SFL = side fluorescence, W = width of dispersion) reached the stability limit within 24 h, most frequently at 37 °C. Lymphocyte size (LY-Z) exceeded the stability limit at 17 h also under storage at 4 °C.

**Conclusions:**

Leukocyte CPD stability is strongly temperature- and parameter-dependent. Exposure to elevated temperature (37 °C) leads to early exceedance of predefined stability limits for several CPD parameters, whereas storage at RT preserves stability for longer time intervals. Avoiding prolonged exposure to high temperatures is therefore essential for reliable CPD assessment in routine laboratory practice.

## Graphical abstract

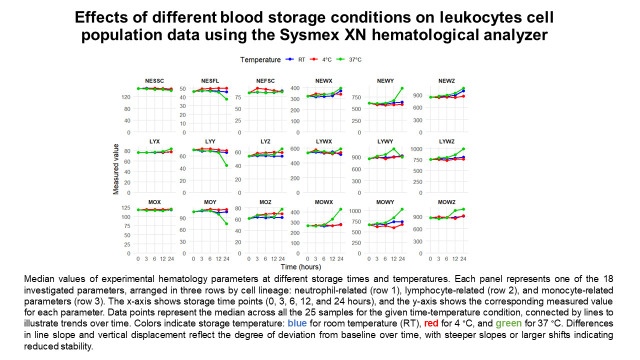

## Highlights

• The present evaluation of 18 experimental hematology parameters under three different storage temperatures consistently demonstrated a predominant pattern of instability at 37 °C, with intermediate changes at 4 °C and the highest stability at room temperature

• Across both the three complementary analytical approaches and the regression-based European Federation of Clinical Chemistry and Laboratory Medicine protocol, this temperature gradient was evident, with several parameters exceeding the maximum permissible error within hours when exposed to heat stress

• Parameters such as neutrophils complexity (NE-SSC), neutrophils size (NE-FSC), monocytes complexity (MO-X), and lymphocytes complexity (LY-X) emerged as consistently stable in both frameworks, maintaining values within stability limits across all conditions up to 24 h

## Introduction

Up to 70% laboratory errors are attributable to extra-analytical issues, especially to inaccurate or inappropriate procedures for management of biological specimens (*1*). The analytical assessment of unsuitable samples represents a major challenge for the quality of diagnostic testing and poses serious hazards to patient safety. The complete blood cell count (CBC) is an essential test for diagnosis and management of a variety of hematological diseases. It is essential that blood samples are always collected, managed, transported and stored under appropriate conditions for obtaining accurate tests results.

Several studies have been published on the stability of whole blood samples for CBC testing with often controversial conclusions, which were mostly attributable to the use of different hematological analyzers (*2*, *3*). Delays in analyzing specimens are not rare occurrences in clinical laboratory practice, because samples may arrive even from very distant peripheral laboratories, or due to technical and organizational issues.

The cell population data (CPD) parameters, that can be obtained from a standard CBC test, have been recently implemented for providing useful information for diagnosis and prognosis in many diseases (*4*-*6*). The CPD describe multiple physical and functional characteristics of leukocytes in the analyzed sample, which may be altered under various pathological conditions, including hematological malignancies, solid tumors, and severe infections (*7*). Nonetheless, preanalytical influences on these parameters have not been comprehensively assessed to the best of our knowledge, while limited evidence has been provided that blood storage may have an impact on their measurement (*7*-*9*). For example, a study by Martens *et al.*, referring to unpublished results, reported that CPD results may be stable up to for 6 hours after sample collection (*10*).

Preanalytical factors remain largely unexplored in CPD research and may represent a critical aspect in obtaining reliable results. The effects of EDTA on blood cell morphology are well documented (*7*, *8*). Since CPD fundamentally reflect cellular morphological and functional features, factors such as storage time and temperature are very likely to significantly influence CPD measurements. Several studies on CPD were retrospective in nature, generating a theoretical risk that poorly controlled preanalytical conditions may have led to inaccurate results and potentially inappropriate conclusions (*7*-*9*).

Therefore, the aim of the study was to investigate the reliability of CPD measured on Sysmex XN hematological analyzer in samples stored for up to 24 h at different temperatures.

## Materials and methods

This prospective study was designed to investigate the influence of preanalytical variables on CPD parameters measured with the XN-3000 series automated hematology analyzer, that employs a technology based on fluorescence flow cytometry (Sysmex XN, Kobe, Japan). The study population consisted of 25 ostensibly healthy subjects. The inclusion criteria were being apparently healthy men or women aged ≥ 18 years.

All blood samples were drawn by a single experienced nurse. For each participant, three evacuated blood tubes containing dipotassium ethylenediamine tetraacetic acid (K2ETDA) (Greiner Bio-One Vacuette, Kremsmunster, Austria) were collected. The filling volume of the test tubes used in this study is 2 mL and we used an integrated venous blood collection systems that comprise a blood collection needle of 19 Gauges, tube holder and the evacuated blood collection tube.

The CPD are automatically measured by XN-3000 and test results are available in a dedicated working window of the instrumental software. All samples were analyzed on the same analyzer using an identical lot of reagents.

The Sysmex XN-3000 hematological analyzer used in this study was calibrated in accordance with the manufacturer’s recommendations and the laboratory’s standard operating procedures. The last full calibration was performed before the initiation of the study, and no recalibration was required during the study period, as instrument performance remained stable. Internal quality control (IQC) was carried out daily at the beginning of each working day using manufacturer-provided control materials (XN-CHECK, Sysmex Corporation) at multiple concentration levels (low, normal, and high). Quality control results were evaluated against the target ranges provided by the manufacturer and monitored using routine laboratory acceptance criteria (including Westgard multirule principles). Throughout the study period, all QC results were within acceptable limits, ensuring the analytical reliability of the measurements. Samples stored under varying conditions were consistently equilibrated to room temperature for approximately 30 minutes prior to analysis, in accordance with the manufacturer’s recommendations.

The values of CPD parameters measured in the current study were: neutrophils complexity (NE-SSC), lymphocytes complexity (LY-X), monocytes complexity (MO-X), width of dispersion of neutrophils complexity (NE-WX), width of dispersion of lymphocytes complexity (LY-WX), width of dispersion of monocytes complexity (MO-WX), neutrophils fluorescence intensity (NE-SFL), lymphocytes fluorescence intensity (LY-Y), monocytes fluorescence intensity (MO-Y), width of dispersion of neutrophils fluorescence (NE-WY), width of dispersion of lymphocytes fluorescence (LY-WY), width of dispersion of monocytes fluorescence (MO-WY), neutrophils size (NE-FSC), lymphocytes size (LY-Z), monocytes size (MO-Z), width of dispersion of neutrophils size (NE-WZ), width of dispersion of lymphocytes size (LY-WZ) and the width of dispersion of monocytes size (MO-WZ) (*11*).

### Cell population data

In the Sysmex XN3000, the white blood cell (WBC) differential count is performed in the white differential channel (WDFc). White blood cells are treated with a lysing reagent that permeabilizes the cell membrane before analysis, followed by staining of deoxyribonucleic acid (DNA) and ribonucleic acid (RNA) with a specific fluorochrome (*11*). The CPD parameters provide quantitative information on the morphological and functional characteristics of neutrophils, monocytes, and lymphocytes, and are derived from the characteristics of the cell clusters identified in the WDFc. Optical signals along the X-axis (side scatter) reflect internal cellular complexity, fluorescence along the Y-axis corresponds to nucleic acid content, and forward scatter (Z-axis) indicates cell size. For each subset, the mean and standard deviation of these parameters are recorded.

The width of dispersion (WDF) of the values is a heterogeneous signal. This parameter is calculated according to the distribution width, as it represents the range of the distribution of the major populations, excluding outliers below 20% peak height in the distribution curve (*10*). The following CPD are reported along the X-axis of the WDFc: NE-SSC, LY-X, MO-X, NE-WX, LY-WX and MO-WX; CPD on the y-axis: NE-SFL, LY-Y, MO-Y, NE-WY, LY-WY and MO-WY; and CPD reported on the z-axis: NE-FSC, LY-Z, MO-Z, NE-WZ, LY-WZ and MO-WZ (*10*-*12*).

### Study design and analytical conditions

For each parameter, whole-blood samples were measured at baseline (T0) and after storage under three temperatures, *i.e.* room temperature (RT), 4 °C, and 37 °C, at four time points: 3 h (T3), 6 h (T6), 12 h (T12), and 24 h (T24). This yielded 12 experimental conditions (3 temperatures × 4 times) plus the baseline. The 37 °C condition was included as an intentional heat stress model to explore the upper limits of temperature-related preanalytical variability, rather than to reflect routine storage practices. Samples assigned to the 37 °C condition were maintained in a thermostatic to ensure stable and reproducible temperature conditions throughout the storage period.

All comparisons were performed within each parameter, with each condition evaluated relative to its corresponding baseline measurement.

### Ethical approval, informed consent and data protection

This study was approved by the Ethics Committee of Milano Area 3 (Reference No. 5725/2025, April 16, 2025) in accordance with the Declaration of Helsinki (2013 revision). Written informed consent was obtained from all study participants and data were processed anonymously in compliance with the General Data Protection Regulation-GDPR EU 679/2016.

### Statistical analysis

Since no established maximum allowable differences were available for these experimental hematology parameters, we adopted three complementary analytical approaches to assess stability.

- Approach 1 - Paired non-parametric testing: for each parameter, we ran 12 paired Wilcoxon signed-rank tests comparing baseline (T0) to each condition (RT/4 °C/37 °C at 3 h, 6 h, 12 h, 24 h). To control the family-wise error rate across the 12 comparisons, Bonferroni’s correction was applied. A condition was classified as non-stable if the adjusted P was < 0.05.
- Approach 2 - Percent difference between medians > 10%: for each parameter and condition, we computed the percent difference between medians of the 25 sample results as PD% = (median at Tx − median at T0) / (median at T0) × 100%. A condition was classified as non-stable if the absolute percent difference between medians was greater than 10%. This threshold was selected as a pragmatic criterion in the absence of established analytical performance specifications for CPD parameters.
- Approach 3 - *Per*-sample percent change thresholding: for each parameter and condition, we computed each sample’s percent change relative to baseline as PD% = (value at Tx − value at T0) / (value at T0) × 100%. We then counted the number of samples with an absolute percent difference between conditions > 10%. A condition was then classified as non-stable when > 1 sample (out of 25) exceeded the 10% threshold (*i.e.* ≥ 2 samples). This criterion was adopted to reduce the influence of isolated outliers and to identify changes affecting more than a single sample, thereby reflecting instability at the population level rather than individual variability.

These approaches provided distinct but complementary perspectives, allowing the identification of parameters and conditions that consistently met or failed the stability criteria (statistical testing and criteria reflecting potential clinical impact). For each parameter, stability was summarized separately by approach (Wilcoxon/medians/*per*-sample) and temperature (RT, 4 °C, 37 °C), considering all time points or only up to 12h.

In addition to the three approaches described above, stability was also evaluated according to the recently proposed European Federation of Clinical Chemistry and Laboratory Medicine (EFLM) protocol for sample stability assessment (*13*). The same convention for percent difference calculation was applied across all analytical approaches to ensure methodological consistency. For each parameter and temperature condition, percentage differences from baseline were calculated for each sample and each time point as: PD% = (Tx - T0) / T0 x 100%, where Tx corresponds to the value of the analyte at 3 h, 6 h, 12 h, and 24 h, while T0 the value at 0 h. This yielded 100 data points per analyte–temperature combination (25 samples × 4 time points). Linear regression was then performed forcing the intercept through the origin, producing estimates of the slope (percentage change per hour), the corresponding p-value, and the coefficient of determination (R^2^). For each analyte–temperature pair, the stability limit was calculated as the time point at which the fitted regression line crossed the maximum permissible error (MPE), set at 10% in this study. This value was selected as a pragmatic threshold in the absence of established analytical performance specifications for CPD parameters and to ensure internal consistency across analytical approaches. A representative example of this EFLM-based stability assessment, including the regression line, MPE threshold, and stability limit, is shown in Figure S1.

The classification followed the adapted EFLM criteria used in this analysis as “Stable”: non-significant slope (P ≥ 0.050), “Unstable”: significant slope (P < 0.050) with R^2^ > 0.500, “To be confirmed”: significant slope (P < 0.050) with R^2^ ≤ 0.500. It should be noted that, in accordance with the EFLM protocol, linear regression was forced through the origin, assuming zero percent difference at baseline. Under this modelling assumption, very small but consistent slopes may reach statistical significance when a large number of observations is available. In such cases, statistical significance reflects the presence of a systematic temporal trend rather than clinically meaningful instability within the investigated time window. Therefore, the estimated stability limit, representing the time required to reach the maximum permissible error, should be considered together with the slope and its significance when interpreting practical sample stability.

All analyses were performed by R software ver. 4.5.1. Comparisons between quantitative variables were evaluated by Wilcoxon signed-rank tests and p values were adjusted using the Bonferroni’s correction.

## Results

The study population consisted of 25 donors (12 males and 13 females), with a median age of 43 (21-56) years.

### Stability assessment using three analytical approaches

The differences from baseline and overall stability were assessed using the three complementary analytical approaches described in the previous Methods section.

Tables 1 and 2 provide a summary of stability assessment for the three analytical approaches. Table 1 includes all time points up to 24 h, whereas Table 2 is restricted to the time window up to 12 h. The latter was designed to highlight stability patterns within time intervals more representative of routine laboratory workflows.

**Table 1:** Summary of stability assessment results for the three analytical approaches across all time points (up to 24 h)

**Parameter**	**Statistical significance** **(P corrected value)**			**%difference between medians > 10%**			**> 2 samples with %difference > 10%**		
	RT	4 °C	37 °C	RT	4 °C	37 °C	RT	4 °C	37 °C
NE-SSC				*	*	*	*	*	*
NE-SFL				*	*				
NE-FSC			*	*		*	*		*
NE-WX					*				
NE-WY	*			*	*				
NE-WZ		*			*				
LY-X				*	*	*	*	*	
LY-Y				*	*		*	*	
LY-Z	*			*			*		
LY-WX	*	*	*	*	*				
LY-WY				*	*				
LY-WZ		*		*	*				
MO-X	*			*	*	*	*	*	*
MO-Y	*			*	*				
MO-Z	*			*					
MO-WX		*		*	*				
MO-WY		*							
MO-WZ	*	*		*	*				
Each row corresponds to one parameter and each column to one of the three approaches, paired Wilcoxon test after Bonferroni correction (P < 0.05), absolute percent difference between medians > 10%, and ≥ 2 samples with an absolute percent change from baseline > 10%, reported separately for each storage temperature (RT, 4 °C, and 37 °C). An asterisk (*) indicates that, for that specific combination of analytical approach and storage temperature, the parameter met the predefined stability criteria at all evaluated time points. Stability criteria were defined as a percentage difference between medians < 10% (Approach 2) and fewer than two samples (out of 25) showing an absolute percent change > 10% (Approach 3). RT - room temperature. NE-SSC - neutrophils complexity. NE-SFL - neutrophils fluorescence intensity. NE-FSC - neutrophils size. NE-WX - width of dispersion of neutrophils complexity. NE-WY - width of dispersion of neutrophils fluorescence. NE-WZ - width of dispersion of neutrophils size. LY-X - lymphocytes complexity. LY-Y - lymphocytes fluorescence intensity. LY-Z - lymphocytes size. LY-WX - width of dispersion of lymphocytes complexity. LY-WY - width of dispersion of lymphocytes fluorescence. LY-WZ - width of dispersion of lymphocytes size. MO-X - monocytes complexity. MO-Y - monocytes fluorescence intensity. MO-Z - monocytes size. MO-WX - width of dispersion of monocytes complexity. MO-WY - width of dispersion of monocytes fluorescence. MO-WZ - width of dispersion of monocytes size.									

**Table 2:** Summary of stability assessment results for the three analytical approaches, considering only time points up to 12 h.

**Parameter**	**Statistical significance** **(P corrected value)**			**%difference between medians > 10%**			**> 2 samples with %difference > 10%**		
	RT	4 °C	37 °C	RT	4 °C	37 °C	RT	4 °C	37 °C
NE-SSC		*		*	*	*	*	*	*
NE-SFL				*	*	*			
NE-FSC	*		*	*		*	*		*
NE-WX	*			*	*	*			
NE-WY	*			*	*	*			
NE-WZ		*		*	*				
LY-X	*			*	*	*	*	*	*
LY-Y				*	*	*	*	*	
LY-Z	*			*		*	*		
LY-WX	*	*	*	*	*	*			
LY-WY		*		*	*				
LY-WZ		*		*	*				
MO-X	*			*	*	*	*	*	*
MO-Y	*			*	*	*			
MO-Z	*			*		*			
MO-WX	*	*		*	*				
MO-WY	*	*		*					
MO-WZ	*	*		*	*				
Each row corresponds to one parameter and each column to one of the three approaches, paired Wilcoxon test after Bonferroni correction (P < 0.05), absolute percent difference between medians > 10%, and ≥ 2 samples with an absolute percent change from baseline > 10%, reported separately for each storage temperature (RT, 4 °C, and 37 °C). An asterisk (*) indicates that, for that specific combination of analytical approach and storage temperature, the parameter met the predefined stability criteria at all evaluated time points. Stability criteria were defined as a percentage difference between medians < 10% (Approach 2) and fewer than two samples (out of 25) showing an absolute percent change > 10% (Approach 3). RT - room temperature. NE-SSC - neutrophils complexity. NE-SFL - neutrophils fluorescence intensity. NE-FSC - neutrophils size. NE-WX - width of dispersion of neutrophils complexity. NE-WY - width of dispersion of neutrophils fluorescence. NE-WZ - width of dispersion of neutrophils size. LY-X - lymphocytes complexity. LY-Y - lymphocytes fluorescence intensity. LY-Z - lymphocytes size. LY-WX - width of dispersion of lymphocytes complexity. LY-WY - width of dispersion of lymphocytes fluorescence. LY-WZ - width of dispersion of lymphocytes size. MO-X - monocytes complexity. MO-Y - monocytes fluorescence intensity. MO-Z - monocytes size. MO-WX - width of dispersion of monocytes complexity. MO-WY - width of dispersion of monocytes fluorescence. MO-WZ - width of dispersion of monocytes size.									

Across the three analytical approaches, no parameter remained entirely stable under all conditions when the full 24 h window was considered. Across the three analytical approaches, NE-SSC, NE-FSC, MO-X, and LY-X met the predefined stability criteria more frequently than other parameters, particularly within the first 12 h (Tables 1, 2).

For these parameters, deviations beyond the 10% threshold or statistically significant Wilcoxon results were infrequent within the first 12 h.

**Figure 1:**
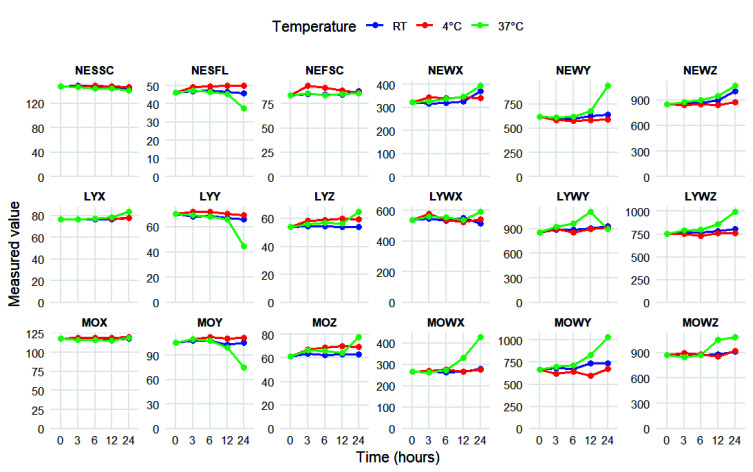
Median values of experimental hematology parameters at different storage times and temperatures. Each panel represents one of the 18 investigated parameters, arranged in three rows by cell lineage: neutrophil-related (row 1), lym-phocyte-related (row 2), and monocyte-related parameters (row 3). The x-axis shows storage time points (0, 3, 6, 12, and 24 hours), and the y-axis shows the corresponding measured value for each parameter. Data points represent the median across all the 25 samples for the given time-temperature condition, connected by lines to illustrate trends over time. Colors indicate storage temperature: blue for room temperature (RT), red for 4 °C, and green for 37 °C. Differences in line slope and vertical displacement reflect the degree of deviation from baseline over time, with steeper slopes or larger shifts indi-cating reduced stability. NESSC - neutrophils complexity. NESFL - neutrophils fluorescence intensity. NEFSC - neutrophils size. NEWX - width of dispersion of neutrophils complexity. NEWY - width of dispersion of neutrophils fluorescence. NEWZ - width of dispersion of neutrophils size. LYX - lymphocytes complexity. LYY - lymphocytes fluorescence intensity. LYZ - lymphocytes size. LYWX - width of dispersion of lymphocytes complexity. LYWY - width of dispersion of lymphocytes fluo-rescence. LYWZ - width of dispersion of lymphocytes size. MOX - monocytes complexity. MOY - monocytes fluorescence intensity. MOZ - monocytes size. MOWX - width of dispersion of monocytes complexity. MOWY - width of dispersion of monocytes fluorescence. MOWZ - width of dispersion of monocytes size.

According to the predefined criteria, a higher number of non-stable conditions was observed at 37 °C compared with 4 °C and RT across the evaluated time points (Tables 1, 2). At room temperature, several parameters met the predefined stability criteria at 24 h according to the applied analytical approaches (Tables 1, 2, Figure 1).

### Stability evaluation using the EFLM protocol

The summary outcomes of this regression-based analysis for all analytes and temperature conditions are presented in Tables 3 and 4, with Table 4 providing an aggregated overview across the entire dataset. Specifically, Table 4 summarizes the EFLM-based stability limits with respect to a 24 h operational time window, indicating whether each parameter exceeded this threshold across all storage temperatures or, when this was not the case, the specific temperature(s) at which the stability limit was reached within 24 h.

**Table 3:** Summary of regression-based stability evaluation according to the EFLM protocol for all 18 experimental hematology parameters under three storage temperatures (room temperature, 4 °C, 37 °C)

**Parameter**	**Condition**	**Slope**	**P**	**Signif**	**R2**	**R2 > 0.7**	**Conclusion**	**SL, h**
NE-SSC	RT	- 0.112	< 0.001	*	0.721	+	Unstable	89
	4 °C	- 0.018	0.087		0.029		Stable	
	37 °C	- 0.235	< 0.001	*	0.842	+	Unstable	43
NE-SFL	RT	0.104	< 0.001	*	0.117		To be confirmed	96
	4 °C	0.530	< 0.001	*	0.618	+/-	Unstable	19
	37 °C	- 0.531	< 0.001	*	0.528	+/-	Unstable	19
NE-FSC	RT	0.120	< 0.001	*	0.210		To be confirmed	83
	4 °C	0.226	< 0.001	*	0.142		To be confirmed	44
	37 °C	0.073	0.010	*	0.066		To be confirmed	136
NE-WX	RT	0.446	< 0.001	*	0.554	+/-	Unstable	22
	4 °C	0.426	< 0.001	*	0.449		To be confirmed	23
	37 °C	0.932	< 0.001	*	0.783	+	Unstable	11
NEWY	RT	0.155	< 0.001	*	0.134		To be confirmed	64
	4 °C	- 0.220	< 0.001	*	0.244		To be confirmed	45
	37 °C	1.838	< 0.001	*	0.640	+/-	Unstable	5
NE-WZ	RT	0.746	< 0.001	*	0.460		To be confirmed	13
	4 °C	0.200	0.023	*	0.051		To be confirmed	50
	37 °C	1.113	< 0.001	*	0.684	+/-	Unstable	9
LY-X	RT	0.065	< 0.001	*	0.389		To be confirmed	154
	4 °C	0.075	< 0.001	*	0.404		To be confirmed	133
	37 °C	0.341	< 0.001	*	0.766	+	Unstable	29
LY-Y	RT	- 0.283	< 0.001	*	0.572	+/-	Unstable	35
	4 °C	- 0.008	0.729		0.001		Stable	
	37 °C	- 1.271	< 0.001	*	0.880	+	Unstable	8
LY-Z	RT	- 0.104	0.122		0.024		Stable	
	4 °C	0.581	< 0.001	*	0.718	+	Unstable	17
	37 °C	0.716	< 0.001	*	0.692	+/-	Unstable	14
LY-WX	RT	- 0.005	0.937		0.000		Stable	
	4 °C	0.096	0.204		0.016		Stable	
	37 °C	0.331	< 0.001	*	0.117		To be confirmed	30
LY-WY	RT	1.682	0.034	*	0.045		To be confirmed	6
	4 °C	0.261	< 0.001	*	0.181		To be confirmed	38
	37 °C	0.799	< 0.001	*	0.253		To be confirmed	13
LYWZ	RT	0.367	< 0.001	*	0.173		To be confirmed	27
	4 °C	0.642	0.316		0.010		Stable	
	37 °C	1.386	< 0.001	*	0.611	+/-	Unstable	7
MO-X	RT	0.011	0.386		0.008		Stable	
	4 °C	0.073	< 0.001	*	0.190		To be confirmed	137
	37 °C	0.021	0.269		0.012		Stable	471
MO-Y	RT	1.887	0.118		0.024		Stable	
	4 °C	2.247	0.066		0.034		Stable	
	37 °C	0.651	0.545		0.004		Stable	
MO-Z	RT	0.078	0.113		0.025		Stable	
	4 °C	0.652	< 0.001	*	0.515	+/-	Unstable	15
	37 °C	0.920	< 0.001	*	0.587	+/-	Unstable	11
MO-WX	RT	0.235	< 0.001	*	0.129		To be confirmed	43
	4 °C	0.234	0.012	*	0.062		To be confirmed	43
	37 °C	2.382	< 0.001	*	0.700	+	Unstable	4
MO-WY	RT	0.597	< 0.001	*	0.213		To be confirmed	17
	4 °C	- 0.146	0.059		0.036		Stable	
	37 °C	2.127	< 0.001	*	0.612	+/-	Unstable	5
MO-WZ	RT	0.620	0.402		0.007		Stable	
	4 °C	0.310	0.012	*	0.062		To be confirmed	32
	37 °C	1.229	< 0.001	*	0.394		To be confirmed	8
For each parameter-temperature combination, the regression slope (% change *per* hour), corresponding P-value, statistical significance (Signif), coefficient of determination (R^2^), and classification outcome (“Stable”, “Unstable”, “To be confirmed”) are reported. The stability limit in hours (SL, h) represents the estimated time to reach the maximum permissible error (MPE = 10%) based on the fitted regression line. In the column “Signif”, “*” indicates a statistically significant slope (P < 0.05). In the column “R^2^ > 0.7”, “+” denotes R^2^ values exceeding 0.7, whereas “±” denotes 0.5 < R^2^ ≤ 0.7 (see EFLM criteria in the text). RT - room temperature. NE-SSC - neutrophils complexity. NE-SFL - neutrophils fluorescence intensity. NE-FSC - neutrophils size. NE-WX - width of dispersion of neutrophils complexity. NE-WY - width of dispersion of neutrophils fluorescence. NE-WZ - width of dispersion of neutrophils size. LY-X - lymphocytes complexity. LY-Y - lymphocytes fluorescence intensity. LY-Z - lymphocytes size. LY-WX - width of dispersion of lymphocytes complexity. LY-WY - width of dispersion of lymphocytes fluorescence. LY-WZ - width of dispersion of lymphocytes size. MO-X - monocytes complexity. MO-Y - monocytes fluorescence intensity. MO-Z - monocytes size. MO-WX - width of dispersion of monocytes complexity. MO-WY - width of dispersion of monocytes fluorescence. MO-WZ - width of dispersion of monocytes size.								

**Table 4:** Aggregated overview of EFLM protocol-based stability classification outcomes across all 18 experimental hematology parameters and three storage temperatures

**Parameter**	**All conditions: Stable, OR Unstable/To be confirmed with limit > 24h**	**≥ 1 condition: Unstable/To be confirmed with limit < 24h**
NE-SSC	X	
NE-SFL		4 °C: 19h, 37 °C: 19h
NE-FSC	X	
NE-WX		RT: 22 h, 4 °C: 23 h, 37 °C: 11 h
NE-WY		37 °C: 5 h
NE-WZ		RT: 13 h, 37 °C: 9 h
LY-X	X	
LY-Y		37 °C: 8 h
LY-Z		4 °C: 17 h, 37 °C: 14 h
LY-WX	X	
LY-WY		37 °C: 13 h
LY-WZ		37 °C: 7 h
MO-X	X	
MO-Y	X	
MO-Z		4 °C: 15 h, 37 °C: 11 h
MO-WX		37 °C: 4 h
MO-WY		RT: 17 h, 37 °C: 5 h
MO-WZ		37 °C: 8 h
For each temperature, the table reports the number and percentage of parameters classified as Stable, Unstable, or To be confirmed. This overall summary provides a comparative perspective on the effect of temperature on stability across the entire dataset. X indicates stability or instability but with limit > 24h. Summary of European Federation of Clinical Chemistry and Laboratory Medicine (EFLM)-based stability limits (SL) with respect to a 24 h operational time window. For each experimental hematology parameter, the first column indicates whether the estimated stability limit exceeded 24 h across all storage temperawtures (SL > 24 h). The second column, populated only for parameters not meeting this criterion, reports the specific storage temperature(s) at which the stability limit was reached within 24 h (SL < 24 h). For parameters listed in the second column, the absence of a given temperature indicates that the stability limit exceeded 24 h under that storage condition. The two columns are mutually exclusive and provide complementary information on the practical time-temperature dependence of parameter stability according to the EFLM protocol. NE-SSC - neutrophils complexity. NE-SFL - neutrophils fluorescence intensity. NE-FSC - neutrophils size. NE-WX - width of dispersion of neutrophils complexity. NE-WY - width of dispersion of neutrophils fluorescence. NE-WZ - width of dispersion of neutrophils size. LY-X - lymphocytes complexity. LY-Y - lymphocytes fluorescence intensity. LY-Z - lymphocytes size. LY-WX - width of dispersion of lymphocytes complexity. LY-WY - width of dispersion of lymphocytes fluorescence. LY-WZ - width of dispersion of lymphocytes size. MO-X - monocytes complexity. MO-Y - monocytes fluorescence intensity. MO-Z - monocytes size. MO-WX - width of dispersion of monocytes complexity. MO-WY - width of dispersion of monocytes fluorescence. MO-WZ - width of dispersion of monocytes size.		

Figure S1 shows a representative example of the EFLM-based stability assessment.

For some analytes, the regression analysis yielded statistically significant slopes associated with estimated stability limits exceeding the maximum investigated storage time (24 h). In these cases, the significant slope reflects the presence of a slow and consistent temporal trend, while the corresponding stability limit indicates that the maximum permissible error would not be reached within the studied time window.

Among the six neutrophil-related parameters, NE-SSC and NE-FSC showed slow rates of change with estimated stability limits exceeding 24 h across all storage conditions, whereas NE-SFL, NE-WX, NE-WY and NE-WZ reached the stability limit within 5-23 h, particularly at 37 °C.

Similar behavior was observed for lymphocyte-related parameters, with LY-X and LY-WX showing stability limits beyond 24 h, while LY-Y, LY-Z, LY-WY and LY-WZ showed progressive deterioration at 37 °C, reaching the stability limit within 7-14 h. LY-Z reached the stability limit at 17 h also when samples were kept at 4 °C, representing the only lymphocyte-related parameter showing instability within 24 h under refrigerated storage. Among the monocyte-related parameters, MO-Z, MO-WX, MO-WY and MO-WZ reached the stability limit within 4-11 h at 37 °C. In contrast, MO-X and MO-Y showed stability limits exceeding the 24 h time window across all storage conditions (Tables 3, 4).

## Discussion

The preanalytical influence on CPD has received little attention in the current scientific literature to the best of our knowledge. Although preanalytical factors remain largely unexplored in CPD research, they are likely to be of substantial clinical significance. Moreover, it is conceivable that the specific technology employed by a hematology analyzer may influence its sensitivity to preanalytical effects, implying that these effects should be evaluated for each analytical platform individually.

Figure 1 shows the temporal profiles of 18 experimental hematology parameters, revealing heterogeneous stability patterns depending on both the analyte and storage temperature. For several parameters, values remained relatively stable over the first 12 hours, particularly at room temperature and 4 °C, whereas storage at 37 °C often resulted in more pronounced changes over time. Some parameters displayed gradual shifts, while others showed abrupt deviations at later time points. The direction of change also varied between parameters, with some increasing and others decreasing compared to baseline. For parameters like NE-SSC and MO-X, the variability remained low up to 12 h across all temperatures.

Taken together, the observed results demonstrated a predominant pattern of instability at 37 °C, with intermediate changes at 4 °C and the highest stability at room temperature.

Across both the three complementary analytical approaches and the regression-based EFLM protocol, this temperature gradient was evident, with several parameters exceeding the maximum permissible error within hours when exposed to heat stress. Parameters such as NE-SSC, NE-FSC, MO-X, and LY-X showed slow rates of change with estimated stability limits exceeding the investigated 24 h time window across storage conditions, indicating preservation of analytical integrity within routine laboratory timeframes. In contrast, several parameters, including NE-SFL, NE-WX, NE-WY, NE-WZ, LY-Y, LY-Z, LY-WY, LY-WZ, MO-Z, MO-WX, MO-WY, and MO-WZ, reached the maximum permissible error within 24 h, particularly under exposure to 37 °C.

When interpreting EFLM-based stability results, statistical significance of the regression slope should not be considered in isolation. In accordance with the EFLM protocol, regression was forced through the origin, which may lead to statistically significant slopes even for very small rates of change when a large number of observations is available. In such cases, the estimated stability limit provides a more informative measure of practical relevance, as it reflects the time required to exceed the predefined maximum permissible error.

In another study the stability of CPD has been assessed. Specifically, Sun *et al* used a Beckman Coulter hematology analyzer for measuring CPD by means of a rather different approach (*i.e*., VCS technology) (*8*). Although their results are not directly comparable to ours due to the use of a fundamentally different analytical approach, they nonetheless warrant discussion. In brief, the study assessed the effects of storing peripheral blood at room temperature on CPD values using samples from 17 healthy donors analyzed up to 48 hours. Neutrophils showed increased mean volume after 8 hours and decreased mean conductivity, suggesting nuclear condensation or cytoplasmic expansion. Light scatter parameters in neutrophils decreased as early as 6 hours, indicating cytoplasmic degranulation and nuclear hypo-segmentation. Lymphocytes exhibited increased mean volume only at 48 hours, while mean conductivity rose earlier, reflecting cytoplasmic contraction. Their light scatter parameters changed within 2-3 hours, suggesting membrane irregularities and chromatin condensation. Monocytes showed stable mean volume and conductivity but significant decreases in light scatter parameters, indicating reduced granularity and nuclear condensation. Standard deviations for all cell types increased over time, reflecting greater population variability. Notably, CPD alterations appeared earlier than morphological changes observed microscopically. Some neutrophil CPD changes mimic pathological conditions such as myelodysplastic syndrome. It was therefore concluded that, consistent with our findings, RT storage significantly impacts leukocyte CPD values in a time- and cell-type-dependent manner.

A key strength of our study is the substantial sample size, the inclusion of four distinct time points, and the comprehensive assessment of 18 parameters across three temperature conditions. To our knowledge, this study is among the few to fully implement the new EFLM stability protocol in parallel with other analytical approaches, enabling direct comparison and methodological validation.

Overall, a clear gradient of instability was observed across all parameter series (RT < 4 °C < 37 °C). Taken together, these findings highlight that samples appear to become instable most rapidly at 37 °C for the majority of the CPD parameters investigated, often within the first 12 h, whilst storage at 4 °C generally delays deterioration but does not fully prevent instability for some parameters. The storage at RT showed the best preservation overall. From a practical standpoint, the key message is that avoiding prolonged exposure to high temperatures is critical to maintaining the integrity of these experimental hematology parameters.

Some limitations of this study should be acknowledged. First, duplicate measurements were not performed at each time point, which may limit the assessment of analytical imprecision over time. Second, the study was conducted on samples from ostensibly healthy subjects, and stability patterns may differ in pathological conditions. Third, the stability assessment was limited to a single analytical platform, and therefore the findings cannot be directly extrapolated to other hematology analyzers employing different technologies. Finally, the 37 °C condition was included as a heat-stress model rather than a routine storage scenario, and its results should be interpreted accordingly. Despite these limitations, the use of multiple complementary analytical approaches and the application of the EFLM protocol provide a robust framework for evaluating CPD stability.

In conclusion, this study demonstrates a clear time- and temperature-dependent instability of leukocyte cell population data (CPD) measured on the Sysmex XN hematological analyzer. Across all analytical approaches, a consistent gradient of instability was observed, with the highest stability at room temperature, intermediate stability at 4 °C, and the most rapid deterioration at 37 °C. Several parameters, including NE-SSC, NE-FSC, MO-X, and LY-X, maintained stability within a 24 h time window across storage conditions, whereas others exceeded predefined stability limits within 24 h, particularly under heat-stress conditions. These findings indicate that CPD are sensitive to preanalytical storage conditions and highlight the importance of controlling temperature exposure to preserve analytical integrity in routine laboratory practice.

## Data Availability

The data that support the findings of this study are available from the corresponding author upon reasonable request.
